# Research training incorporating education and mentoring for rural and regional allied health professionals: An evaluation study

**DOI:** 10.1111/ajr.12879

**Published:** 2022-05-21

**Authors:** Olivia A. King, Anna Wong Shee, Owen Howlett, Renee Clapham, Vincent L. Versace

**Affiliations:** ^1^ Barwon Health Geelong Victoria Australia; ^2^ South West Healthcare Warrnambool Victoria Australia; ^3^ Monash Centre for Scholarship in Health Education Clayton Victoria Australia; ^4^ Ballarat Health Services Ballarat Victoria Australia; ^5^ Deakin University Warrnambool Victoria Australia; ^6^ Bendigo Health Bendigo Victoria Australia; ^7^ La Trobe University Rural Health School Flora Hill Victoria Australia

**Keywords:** allied health professionals, research capacity building, research mentoring, research training

## Abstract

**Introduction and Objective:**

Building rural health workforce research capacity is critical to addressing rural health inequalities. Research training is a mainstay research capacity building strategy. This paper describes the delivery and evaluation of a research training program for rural and regional allied health professions (AHPs).

**Design:**

A mentored research training program was delivered to AHPs employed public health services in rural and regional Victoria, Australia. The program was evaluated using the Evidence‐Based Practice Knowledge Attitudes and Practice (EBP‐KAP) tool at baseline and 3 months post‐training. Semi‐structured interviews undertaken at 3 and 16 months post‐training explored participants' perspectives of the training, their development and application of EBP and research skills. Survey data were analysed descriptively, and interview data were analysed using a framework approach.

**Findings:**

Thirty‐four individuals from 14 organisations attended the first workshop and 31 attended the second. Thirty‐one participants completed the survey at baseline and nine at 3 months post‐training. Sixteen interviews were undertaken with 11 participants, five participating at both time points. Participants had positive EBP attitudes at both time points. Overall, participants' knowledge and incorporation of EBP into their practice, and retrieval of evidence was unchanged 3 months post‐training. Themes identified in the interview data were as follows: (1) individual research capacity enhanced through supported practice, (2) organisational factors influence individuals' progression of research and (3) individual contributions towards research capacity within the organisation.

**Conclusion:**

A mentored rural research training program promoted the application of EBP skills at the individual level and contributed to organisational research capacity.

**What is already known on this subject:**

- Research led by health professionals and health services influences evidence‐based practice, improves service‐user outcomes, and health workforce recruitment and retention
- Rural health services and health professionals have less access to research capacity building initiatives and opportunities than those in metropolitan areas
- Investment in rural health research capacity building is a key policy directive and strategy to reduce geographic location‐related health disparities

**What this study adds:**

- A mentored and applied research training for allied health professionals led to several individual outcomes beyond increased research and evidence‐based practice knowledge. It led to the development of rural research networks, new opportunities and ways of thinking about and approaching clinical practice changes
- Organisational research capacity and culture influenced the outcomes for individual allied health professionals that engaged in research training
- Although targeted at the individual allied health professional level, the training impacted on organisation‐level research capacity

## 1 INTRODUCTION

Allied health incorporates numerous disciplines such as occupational therapy, physiotherapy and dietetics.^1^ AHPs represent the second largest component of the health workforce and play a key role in optimising health consumer outcomes.^2^, ^3^ AHPs are motivated to engage in research to generate research evidence that will improve the delivery of care and health outcomes; however, they are less likely to be engaged in research than medical professionals and require training and ongoing support to move through the various phases of research.^4^, ^5^, ^6^ This is particularly the case for novice AHP researchers in rural areas who face unique challenges related to chronic workforce shortages, and professional and geographic isolation.^7^

Embedding research as core business in health services is essential for the delivery of evidence‐based practice (EBP) and effective health care that improves service‐user outcomes, and promotes health workforce recruitment and retention.^8^, ^9^, ^10^, ^11^, ^12^ Promoting health service‐led research in rural areas ensures relevant evidence is generated to improve service delivery and the health of rural communities.^7^, ^8^, ^9^, ^13^, ^14^ Rural health services and AHPs, however, have less access to research infrastructure, research capacity building (RCB) initiatives and research funding and therefore fewer opportunities to lead and participate in research than their metropolitan counterparts.^5^, ^7^, ^8^, ^14^, ^15^ Alongside health policy‐makers in other Australian states and territories, the Department of Health in Victoria has committed substantial investment into resources and infrastructure to engage AHPs in research and build research capacity.^16^, ^17^, ^18^

In Victoria, Allied health incorporates 27 professions, including disciplines such as occupational therapy, physiotherapy and dietetics.^1^ AHPs represent one third of the health workforce and play a key role in optimising health consumer outcomes.^2^ AHPs are motivated to engage in research to generate research evidence that will improve the delivery of care and health outcomes; however, they require training and ongoing support to move through the various phases of research.^4^, ^5^ This is particularly the case for novice AHP researchers in rural areas, which includes inner regional, outer regional and remote areas.^19^ Rural AHPs face unique challenges related to chronic workforce shortages, and professional and geographic isolation.^7^

Promoting health service‐led research in rural areas is critical to ensuring relevant evidence is generated to improve service delivery and, in turn, the health outcomes of people living in these communities.^7^, ^8^, ^9^, ^13^, ^14^ Rural health services and health professionals, however, have less access to research infrastructure, research capacity building (RCB) initiatives and research funding than their metropolitan counterparts. Therefore, they have comparatively fewer opportunities to lead and participate in research.^5^, ^7^, ^8^, ^14^, ^15^ Indeed, it has been identified that strategies to develop a safe, high‐quality and sustainable rural and regional health system in Victoria must encompass enhanced research infrastructure in these areas.^20^ For example, establishing research positions and partnerships between health services and research institutions.^13^ A report published in 2020 on the evaluation of the national RHMT Program^21^ outlined a series of key recommendations including the following:

‘Recommendation 15 Through the RHMT program, universities be required to demonstrate that they are supporting rural research through the RCS and UDRH network by:

Delivering high‐quality research training, skills development and research support to local health professionals, supervisors, students and broader community stakeholders

Developing regional consultative mechanisms to identify and respond to local research needs’.ref. 21, p. 28

Effective RCB initiatives encompass a range of integrated strategies targeted at the policy or systems, organisational, team and individual level.^5^, ^7^, ^22^, ^23^ At the individual level, education, training and mentoring are the cornerstone components of RCB initiatives.^24^ Training programs, if available at all in rural contexts, are unlikely to be informed by local needs^7^ and tend to be ad hoc, fragmented and unsustainable.^7^, ^8^ Calls have been made for integrated research training and education programs that are developed and delivered by local researchers and educators for rural health professionals and health organisations.^7^, ^21^, ^25^

This paper describes a research training program developed and delivered in a rural setting to AHPs and the outcomes of the evaluation.

### 1.1 Aims

This evaluation study aimed to:

1. measure the effect of the research training program on participants' knowledge, attitudes and practice regarding evidence‐based practice;
2. explore the outcomes and impacts (if any) of the research training program on participants' individual EBP and research activity;
3. explore the factors that influenced the outcomes and impacts of the training program on individual EBP and research activity; and
4. explore any potential influence of the research training program on research capacity within participants' health organisations.

## 2 METHODS

### 2.1 The turning ideas into proposals research training program

To address current evidence and practice gaps, a research training program targeting point‐of‐care AHPs in rural and regional health settings was developed in 2019. AHPs from 27 public health organisations located in rural and regional settings^26^ in western Victoria, Australia, were invited to submit an expression of interest. Across Victoria, Modified Monash Model areas 2–5 (regional through to small rural towns) comprise almost 23% of the state's population.^27^

The overarching aim of the program was to support novice researchers to enhance participants' EBP knowledge, skills and attitudes and to develop a small‐scale study protocol relevant to their practice setting. Training consisted of two face‐to‐face full‐day workshops at a rural health service held 6 weeks apart, with mentoring by experienced researchers, between workshops. It was anticipated that the protocols developed throughout the training would be submitted as part of research ethics applications to enable participants to progress their research activity.

Active pedagogies were employed in both training workshops to maximise participants' engagement with the training content and its application to their identified research problem during, between and beyond the workshops.^28^ Expressions of interest (EOIs) needed to identify a practice‐related issue to promote *close to practice* research skills.^23^ EOIs were signed by the lead applicant's manager to ensure a minimum level of organisational support. The first workshop included a peer‐to‐peer presentation of research ideas, formulating a researchable question, structuring and undertaking a systematic literature search, using program logic to inform the research design and data collection methods. Workshop two included a follow‐up peer‐to‐peer presentation of research ideas, interview and focus group guide development, survey and audit tool development workshops and a research ethics session.

### 2.2 Study design

A sequential mixed‐methods study design was used to address the aims of the study. Although the workshop aimed to support participants to develop a research protocol as a tangible outcome, the evaluation focused on measuring the development and application of EBP skills through the quantitative survey and used interviews to explore more deeply, the outcomes and application of EBP in health settings. All AHPs who participated in the research training program were invited to take part in the evaluation.

### 2.3 Data collection

To quantitatively measure participants' EBP knowledge, attitudes and practice, the 23‐item EBP‐Knowledge Attitudes Practice (EBP‐KAP)^29^ survey was administered. The 23‐item EBP‐KAP is a modified version of the validated 43‐tem knowledge, attitude and behaviour survey that was developed and widely used for undergraduate health professions education.^30^, ^31^ The 23‐item EBP‐KAP was developed to assess EBP‐related attitudes and practices alongside knowledge and skills, key constructs for understanding behaviour change. EBP assessment tools often focus on specific knowledge components or technical skills. However, attitudinal, perceptual and behavioural factors are important potential barriers to the adoption of EBP. The EBP‐KAP survey assesses 23 research items that a registered nurse or other health professional might encounter in clinical practice, including the utilisation and conduct of research. Each item is measured via a 6‐point Likert scale. There are four subscales within the tool: (1) knowledge; (2) attitudes; (3) information retrieval practices; and (4) professional practice and learning (ref. 29, p. 157). The EBP‐KAP has previously been demonstrated to be a sufficiently reliable and valid tool for measuring differences between the different but related constructs, knowledge, attitudes and practices (information retrieval practices; professional practice and learning) related to EBP in health care professional practice.

The survey was administered in hard copy form at baseline (prior to the training) and then again electronically 3 months post‐training.

Semi‐structured telephone interviews were conducted by a researcher (OK) who has extensive qualitative interview and research experience and was not involved in the delivery of the training. The interviews explored the outcomes and impacts of the training program more broadly and to understand whether the training or other factors influenced participants' research and EBP capacity development, the application of these skills, research progress and any other organisational impacts (see Data S1 Interview guide). Twenty‐nine training attendees consented to being contacted by a member of the research team after the training for an interview.

Interviews were conducted at two time points: (1) August 2019 (3 months post‐training) and (2) September 2020 (16 months post‐training). The 12‐month interviews were delayed due to COVID‐19‐related disruptions. The semi‐structured interview guide was informed by the RCB literature, the researchers' knowledge and experiences of the intended outcomes of research training: the improvement in, and application of participants' EBP and research skills. Interviews were audio‐recorded and professionally transcribed verbatim.

### 2.4 Data analysis

Survey data were analysed using IBM SPSS Statistics (version 27). Mean scores for the knowledge, attitudes and practices subscales were calculated by calculating the individual mean score for the subscale and then taking an average of those scores as a summary measure for the whole group. Negatively worded items were reverse scored for subscale score calculations. Differences on the knowledge, attitudes and practices subscales baseline scores were analysed using a within‐subjects ANOVA. Differences in knowledge, attitudes and practices subscales pre‐ and post‐training were analysed using a Wilcoxon signed rank test.

Interview data (3 months post‐training) were analysed according to a team‐based framework thematic analysis approach, using both inductive and deductive techniques.^32^ After familiarising themselves with the data, three members of the research team (OK, OH, RC) conducted an initial inductive analysis of half of the transcripts from the 3‐month post‐training interviews, each. These analyses identified potential themes and codes, and along with Cooke's framework for evaluating RCB activities^23^ contributed to the initial coding framework, which the three authors reviewed before one of the authors (OH) used it to code the interview data using NVivo11. Two authors (OH and OK) coded the 16‐month post‐training interviews, identifying several new codes, and patterns across all interview data. A summary report was developed and reviewed by all members of the research team.

Quantitative and qualitative data were analysed separately. Analysis of the survey results (i.e. changed in EBP knowledge, attitudes and practice) was completed prior to the interviews. Findings from the survey were explored in greater depth through the interviews. The interview questions were determined prior to the completion of the quantitative component.

## 3 RESULTS

The training program included 13 project teams from 14 health organisations, with 34 participants attending the first workshop and 31 the second workshop.

### 3.1 Surveys

Thirty‐one participants (91.2%) completed the 23‐item EBP‐KAP survey at baseline (Table 1), and nine (26.5%) completed the survey at 3 months post‐training. Most of the respondents were female (*n* = 33, 82.5%). Half of the participants were aged between 30 and 39 years (range 20–60+ years). A third of participants had been working for <5 years. Thirteen disciplines were represented including nine Allied Health professions. Within‐subjects' comparisons of the subscale scores resulted in a significant main effect (*F* (3) = 13.068, *p* < 0.001). Respondents' scores on knowledge (M = 5.14, SD = 0.46) were significantly higher than scores on both information retrieval practices (M = 4.48, SD = 0.62, *p* < 0.001) and professional practice and learning (M = 4.65, SD = 0.49, *p* < 0.001) subscales. The two practice subscales were not significantly different from one another (*p* = 1.000). The knowledge and attitudes (M = 4.92, SD = 0.51) were not significantly different from one another (*p* = 0.378). The attitudes subscale mean score was significantly different from information retrieval practices (*p* = 0.028), however not significantly different from the professional practice and learning subscale (*p* = 0.055).

**Table 1:** 23‐item EBP‐KAP baseline survey results (*n* = 31)

Factor (range)	Item	Mean score (SD)
Knowledge		5.14 (0.46)
	1. Clear understanding of what evidence‐based practice is	4.93 (0.68)
	2. EBP increases efficacy	5.19 (0.56)
	3. Formulation of relevant questions	5.15 (0.53)
	4. Searching skills required	5.19 (0.83)
	5. Assess quality of research	5.41 (0.64)
	6. Integrate with professional practice	4.96 (0.76)
Practice—Information Retrieval		4.49 (0.62)
	7. Access evidence	4.74 (0.71)
	8. Access online sources	4.78 (0.89)
	9. Primary sources of evidence	4.33 (0.96)
	10. Systematic reviews	4.07 (0.83)
Professional practice and learning		4.65 (0.49)
	11. EBP is part of my learning	4.56 (0.80)
	12. EBP positively affects practice	5.04 (0.59)
	13.EBP part of teaching in practice settings	4.56 (0.75)
	14. Doing EBP changed how I learn	4.44 (0.75)
Attitudes about EBP		4.92 (0.51)
	15. EBP will become standard practice	5.07 (0.68)
	16. appreciate advantages of EBP	5.04 (0.65)
	17. EBP disregards professional experience^a^	2.93 (0.87)
	18. EBP will not last, no need to do it^a^	1.70 (0.78)
	19. EBP ignores art of my work^a^	2.07 (0.96)
	20. Work is about helping people, not statistics^a^	1.44 (0.51)
	21. My experience is more important^a^	2.52 (0.80)

*Note:* Abbreviated wording of items provided.

a Items are negatively worded and were reverse scored before calculating subscale scores.

#### 3.1.1 Pre–post analysis

A small number of participants (*n* = 9, 26.5%) completed both the baseline and 3‐month surveys. There were no significant differences in mean scores for the factors: knowledge pre‐analysis (M = 5.15, SD = 0.38) and post‐analysis (M = 5.43, SD = 0.39; *z* = 1.13, *p* = 0.257), practice information retrieval pre‐analysis (M = 4.61, SD = 0.73) and post‐analysis (M = 4.89, SD = 0.52; *z* = 1.16, *p* = 0.246), professional practice and learning pre‐analysis (M = 4.72, SD = 0.23) and post‐analysis (M = 4.86, SD = 0.49; *z* = 0.85, *p* = 0.395), and attitudes about EBP (M = 4.86, SD = 0.46) and post‐analysis (M = 5.15, SD = 0.40; *z* = 1.77, *p* = 0.76). This pre–post analysis is under powered due to the small sample size.

### 3.2 Interviews

Eight semi‐structured interviews were conducted at each time point (total of 16 interviews), with 11 participants. Interviews lasted between 11 and 30 min in duration. Five participated at both the 3‐ and 16‐month time points. Through thematic framework analysis of the combined three‐ and 16‐month post‐training interview data, we identified three key themes: (1) individual research capacity enhanced through supported practice, (2) organisational factors influence individuals' progression of research and (3) individual contributions towards research capacity within the organisation.

#### 3.2.1 Individual research capacity enhanced through supported practice

The outcomes of the training program described by participants varied from ceased project initiatives to finalised study protocols. Of the interview participants representing 11 teams, four had developed a study protocol by the 16‐month post‐training timepoint, two reported that their protocols were undergoing ethics and governance review, one study was in progress, and one had completed their study and were now preparing a manuscript. Despite the variation in terms of progress, participants appreciated gaining basic research skills and an understanding of ‘how to get started’ on their research journey, and there was a sense that the research training provided much more than research knowledge and skills. The practical nature of the training and interactive learning environment provided opportunities for participants to apply the workshop content, using their own research idea, so that it was able to be taken forward in a structured and systematic way. Through practical application of research knowledge, participants were able to experience and demystify the research process:

realising that research is achievable and it's not just something that imaginary people do at universities. So seeing it as being something that clinicians can do and that you don't need special powers. Obviously you need support to be able to do it, but that is a realistic goalParticipant 2, 16 months post‐training

References to the practical, applied nature of the training were made frequently and described as a valued feature of the program. Linked to this were the social and collaborative characteristics of the workshops, where participants shared and contributed to one another's projects. This facilitated peer learning, lateral thinking and the refinement of research ideas

It's great to see other people's designs and ideas and some of their input … questions were really helpful because things that were clear in our minds were clearly not understandable to people who were not embedded in our project the same way we were.Participant 5, three months post‐training

The mentoring component of the training further promoted the practical application of the content and was a frequently reported and somewhat novel highlight for participants:

they [mentor] were highly qualified … super supportive and super enthusiastic that made us more enthusiastic about the project which helped … She was just there to help … she gets back to you as quickly as possible. You felt really supported. That's something that you don't get ever really.Participant 2, three months post‐training

The practical nature of the training program coupled with the mentoring meant participants could apply the knowledge and skills they learned in the workshops and guided them through the challenges faced between and beyond the workshop context. In this sense, the workshops provided the starting point or springboard for participants. Although it was not an expectation of the program, mentorship extended beyond the defined training period for some and enabled the novice researchers to continue journey in a support way.

#### 3.2.2 Organisational factors influence individuals' progression of research

On describing the factors that influenced the progress of their research project, participants referred to a range of organisational factors including team‐related factors, research infrastructure and organisational research culture. Participants referred to supportive managers and team leaders as key factors enabling and promoting research progress:

[Health Organisation] is very supportive of clinical research and they've got [Name] as the research manager, so it's just part of the culture here as well about researching and new program development and that's just very well supported.Participant 10, three months post‐training

An enabler to their research progression was their ease of access to the helpful health service embedded researcher for guidance; however, the same participant described a barrier to the progress was the lack of response from the person required to support and sign‐off on their ethics application. The same participant describes several other organisational influences on research progress:

we haven't even got it [ethics application] in yet. I've sought [Embedded researcher's] support to try and get some information about the next steps. [Embedded researcher] was really good, but it went to the next person at the hospital and I can't get people to call me back or answer my emails … after a few attempts, I stop and then it goes off my radar again.Participant 10, three months post‐training

The above participant quote illustrates how organisational factors and barriers combined with limited time for AHPs to dedicate to research result in research endeavours being abandoned. A lack of time and competing priorities are well‐documented barriers to health professional‐led research, and this was further reinforced by data generated in this study.

COVID‐19 negatively impacted the progress of each of the 16‐month interview participants' research projects, due to staff being redeployed to manage outbreaks and related changes to clinical programs and practice. Nonetheless, this next participant describes a creative approach to providing time to be allocated to facilitate research progress:

She [manager] was definitely supportive of us quarantining some time and extending our supervision time that we usually have from a clinical perspective, to also incorporate some research time … that's an enabler, otherwise I don't think we would have got there.Participant 5, three months post‐training

In the case described above, the manager provided the participant with the scope to be flexible in their use of clinical supervision time and allowed them to dedicate that ‘quarantined’ time to their research. On the flip side, some participants felt that research was not valued by their organisation, which limited their ability to dedicate the time required to progress their research idea:

One of our clinicians' hours got reduced which meant then she had more time available to ‐ in her own time to work the other project, which is not ideal … the barriers are definitely getting approval to do that in your work time and for the organisation to see it as a priority.Participant 2, 16 months post‐training

Other organisation‐related factors included staff turnover which led to the ‘stop‐starting’ of research projects:

all of our research work has been put on hold. Following the end of the training, it was [put] on hold because we had shifting priorities due to staff members leaving, so we were redistributing all of the projects.Participant 8, 16 months post‐training

Staffing shortages are a recognised barrier to progressing new health service initiatives; however, this is arguably more critical in rural and regional health services where it is more difficult to recruit and retain staff.

#### 3.2.3 Individual contributions towards research capacity within the organisation

The training was designed as an RCB initiative targeted at the individual level; however, the interview data indicated that individuals were contributing their organisations' research capacity. This was evident in several ways. The social learning environment provided opportunities for participants to connect with peers from within their organisation and across the region, and some of those wider connections were then sustained post‐training:

it [training] was a really good chance to network and liaise with other clinicians across different health settings, who are working on similar projects, or even working on completely different projects. Just to know that those other research projects are happening and are out there … One of the people that we were meeting with on the day actually has ended up being involved in our study.Participant 3, three months post‐training

The above quote illustrates that training is about more than research knowledge, skills and outputs, and it is also about establishing and fostering research networks, which are pivotal for all novice researchers, arguably even more so for researchers working in organisations that have less research infrastructure or an immature research culture.

In other cases, managers were able to fully appreciate the benefits of the training on individual team members that participated, and took action to embed research in their core business:

My manager is really excited to keep doing research now that we've done it once and he said if you come back next year then we would be really keen to write that into your project plan and KPIs [key performance indicators], so there's definitely potential there.Participant 6, 16 months post‐training

Research training focused on the individual level makes research ‘accessible’ for novice AHP researcher and has the potential to light ‘a spark’ in some:

making that research scene accessible just for your average clinician. I know particularly for that one member in our team, she's seriously considering whether she goes and does further research and does her PhD. So, for her it really lit a spark.Participant 2, 16 months post‐training

This illustrates that providing opportunities for individuals to access and participate in research activity can be the first step in longer‐term and higher‐level engagement with research. A less frequent, yet important outcome for participants was the identification of health service research employment opportunities. For this next participant, having ‘that mindset about research being an accessible thing, I have actually just applied for a research assistant position at my workplace’ (Participant 2, 16 months post‐training). This once again speaks to the development of organisational research infrastructure including a positive research capacity and career structure for AHP researchers.

Several participants reported sharing their learnings from the research training with colleagues within their teams and organisations. A combination of informal or ad hoc opportunities to share their learnings, and more structured inservices and presentations at team meetings were described. For example, this next participant has taken multiple opportunities to share their learnings with their team and wider network:

I have done two in‐services to our OT department since the training scheme. We've just recently applied to be part of our annual research symposium that's happening this year.Participant 5, 16 months post‐training

The quote above illustrates how individual RCB directly influences the organisational capacity and, indeed, the development of team and organisational research resources and infrastructure.

## 4 DISCUSSION

The *Turning Ideas into Proposals* research training program aimed to facilitate novice researcher participants' completion of a research protocol, and in the process, improve their EBP knowledge, attitudes and practices. Although these aims were not fully achieved, due to multiple factors including COVID‐19‐related disruptions, the interview data illustrated that the training program led to more important and yet less quantifiable impacts. It is recognised that ‘stand‐alone’ research training is limited in its ability to produce tangible research outputs, particularly where organisational research capacity and access to research resources, is a local challenge.^22^, ^33^ Moreover, a perennial challenge for those concerned with RCB is demonstrating longer‐term and well‐defined impacts of their programs beyond traditional research metrics.^15^

The individual‐level outcomes of the training are demonstrated via the EBP‐KAP, which indicated that training participants had positive attitudes towards EBP at both baseline and 3 months post‐training. These positive attitudes are not unexpected, given that training participants prepared and submitted an expression of interest for the training program, secured manager approval and took 2 days out of their regular work, with many travelling considerable distance to participate. The significant differences between AHPs' knowledge and attitudes, compared with their evidence‐based practices (information retrieval and professional practice and learning), were similar to those reported in a study evaluating EBP in nursing and AHP students.^29^ Although knowledge and attitudes do not in themselves predict behaviour,^34^ there is evidence that these constructs make a substantial contribution to the adoption of EBP.^35^, ^36^ EBP and research training interventions for health professionals are complex, due to the clinical behaviours, attitudes and knowledge needed for success. It is important that research training has a focus on these factors that build health professional capability and confidence to use EBP in practice.

Participants' knowledge of EBP, incorporation of EBP into their professional practice and retrieval of evidence were also unchanged at the three‐month post‐training mark. Other authors have noted imperceptible changes to participants' EBP knowledge and practices post‐RCB intervention and attributed this to the short duration of the intervention.^37^ In the present study, the lack of a significant change in self‐reported EBP domains is likely due to the small sample size and the brief implementation approach. Attempts to change AHPs' EBP is challenging in a short time frame, and the aims of the program were ambitious given the training format and content. Initiatives for RCB and promoting EBP need to have well planned multi‐faceted implementation approaches for long‐term success. Finally, given that EBP knowledge, attitudes and skills did not change in either direction, this might suggest a ceiling effect.^38^ Overall, findings from our survey and interviews highlight that when measuring EBP outcomes, it should not be assumed that knowledge and attitudes are proxy measures for AHPs' evidence‐based practices.

The interviews enabled exploration of individuals' application of their EBP skills in their research endeavours and clinical practice. It was found that the training‐related factors that most cogently influenced AHPs' confidence to embark on research activity and utilise research to guide clinical practice (i.e. engage in EBP) were the practical nature of the training, and the support of an experienced and accessible research mentor. Indeed, the evaluation highlighted that some mentorships continued beyond formal training period, despite this not being expected of the mentors or the program. The value that experienced and accessible research mentors bring to the novice researcher journey is well‐established and now considered core component of individual‐level RCB strategies.^4^, ^22^, ^39^

Similarly, the importance of the social, collaborative nature of the workshop activities, the peer connection and networks facilitated by the training cannot be underestimated. This social connection aspect is particularly important for novice researchers and those in rural and regional settings.^33^, ^40^, ^41^ In their evaluation of a rurally based novice researcher training program, Duncanson et al.^40^ found that the peer support and peer learning elements of their training program were highly valued and contributed to participants' increased confidence and motivation to continue to engage in research activity. Other authors have reported on the benefits of peer learning and support featured in novice researcher training programs.^42^, ^43^, ^44^

Nonetheless, organisational factors seemed to supersede the practical nature of the training with respect to influencing the outcomes and impacts of the training. This was evident in both the positive and negative sense. Through the expression of interest process, manager support for the participants' research ideas was evident. When managerial and organisational support was combined with key research resources, for example access to embedded researchers and other research infrastructure, participants described their ability to progress their research to varying extents. The opposite was apparent in the absence of organisational support and infrastructure. There is a need for greater engagement with managers and organisations in the early stages of the training program and throughout, to support, progress and sustain AHP‐led research activity. Further, there might be merit in supporting potential AHP research training participants to identify organisational barriers to conducting research and to address these pre‐emptively, for example by using the Capability, Opportunity, Motivation—Behaviour Framework.^45^

The importance of organisational research culture and context on individual research capacity is well‐established^7^, ^22^, ^23^, ^33^, ^39^, ^41^, ^46^; however, the evaluation of the research training program suggests a bi‐directional relationship between individual and organisational RCB strategies. The individually targeted training promoted the sharing of new knowledge with colleagues, which might provide an impetus for them to embark on a research project. One participant was able to demonstrate the value of AHP‐led research activity to managers who in turn acted to embed research activity into key performance indicators, an example of a direct contribution to the development of organisational infrastructure.^47^ The establishment and fostering of regional research networks are further examples of the means by which individuals influence the organisational and broader research context, as previously documented in the RCB literature.^22^, ^39^, ^41^ Although sustainable and impactful RCB strategies must address multiple levels of influence, the findings of the current research training reinforce that for rural and regional AHPs, research education, training and mentoring contribute to both individual‐ and organisation‐level RCB. Cooke et al. point to the *interrelatedness* of RCB strategies and levels of influence, and call on those concerned with RCB appreciate the unquantifiable benefits of providing a ‘catalyst for releasing potential research energies from within individuals and organisations’ (ref. 22, p. 17).

Numerous policy directives highlight the need to bolster research infrastructure in rural and regional areas, implement rural research training programs and establish strategic research partnerships to support capacity building rural and regional areas and promote research that addresses local needs.^13^, ^17^, ^21^ The *Turning Ideas into Proposals* research training program is a low resource‐intensive program when compared with other rural research capacity building programs (e.g. refs 40, 48) that has contributed to the realisation of some of these key aspirations. It provides a useful blueprint for organisations concerned with rural health RCB and other initiatives to strengthen rural health workforces. Indeed, it has informed the development of a larger regional research training program that focuses on all health practitioner groups, not just AHPs.

This evaluation study is limited by the small response to the EBP‐KAP survey post‐training and the lack of a third timepoint for the EBP‐KAP survey. The planned EBP‐KAP survey at the 16‐month post‐training evaluation mark was not conducted due, in part, to the impact of COVID‐19 in health organisations. Further, that the perceived outcomes and impacts were explored from the perspectives of the training participants only, and the perspectives of health organisation managers, executives, mentors and health care consumers were not captured.

Future research to explore any differences in the experiences of the different allied health professions in developing and applying research and EBP skills in their health service setting would be enlightening.

## 5 CONCLUSION

*Turning Ideas into Proposals* is a practical research training program that was developed with rural AHPs in mind and delivered in a rural health setting. The impact on participants' overall EBP knowledge, attitudes and practices was modest; however, the evaluation highlights the training program's influence on the application their EBP and research skills in the research endeavours and clinical practice. Although these outcomes were described at the individual participant level, considering the interaction between individuals' enhanced knowledge and skills and their organisational context and infrastructure, the training program represents a catalyst for promoting a research culture among rural AHPs in Victoria. Further investment in locally developed regional and rural health professional research education and mentored training is warranted to promote the development of individual and organisational research capacity and to sustain and progress these positive outcomes. The success and sustainability of such programs for participants are dependent, at least in part, on the engagement of managers and organisations both during and beyond the program EOI stage. The findings of our evaluation have informed the development of a broader program of research capacity and capability building in rural and regional Victoria.

## AUTHOR CONTRIBUTIONS

OAK: conceptualization; data curation; formal analysis; methodology; project administration; writing – original draft; writing – review and editing. AWS: conceptualization; data curation; formal analysis; funding acquisition; methodology; project administration; writing – original draft; writing – review and editing. OH: conceptualization; formal analysis; methodology; writing – original draft; writing – review and editing. RC: conceptualization; formal analysis; methodology; writing – original draft; writing – review and editing. VLV: conceptualization; methodology; writing – original draft; writing – review and editing.

## CONFLICTS OF INTEREST

The authors declared they have no conflicts of interest.

## ETHICS APPROVAL

Ballarat Health Services Human Research Ethics Committee granted approval (51884).

## Supporting information


Data S1
