# Supplementary material for: Research training incorporating education and mentoring for rural and regional allied health professionals: An evaluation study
Source: Aust J Rural Health. 2022 May 21;30(5):654–65. doi: 10.1111/ajr.12879 (PMC9790691; doi:10.1111/ajr.12879)
Supplement: Supplementary file 1 — Data S1 [file AJR-30-654-s001.docx]

## Turning Ideas into Proposals Training Scheme: Evaluation

## Interview guide

#### Learning

Since commencing the Turning Ideas into Proposal Training Scheme, which comprised of the two workshops (in March and May 2019), mentoring between workshops and protocol development; how have your research and clinical practice skills changed or improved?

Thinking about your experience of the Training Scheme and developing your project, have there been any unexpected learnings or outcomes?

#### Behaviour

Which particular skills or learnings from the Training Scheme have you applied or used in your research or clinical work?

Have you accessed any other research training during or since the Training Scheme?

Have you shared your learnings with others in your team or health service either formally or informally?

In what way/s have you or do you use the skills and knowledge you gained during the Turning Ideas into Proposals Training Scheme?

*Has the way you think about or approach changes to clinical practice changed?*

*Have you shared the findings of your research project (e.g. via publication or presentation)?*

*Have you applied the learnings to different projects?*

If you have not yet completed a research protocol, or converted the protocol into a research project, what do you identify as being the main barriers?

*What would be needed for you and your workplace to apply the knowledge in practice?*

*OR*

*What have been the enablers?*

Do you have any other comments, recommendations or anything else to add about the Turning Ideas into Proposals Training Scheme or research education in general?

Thank you
